# Engaging medical and dental students in curriculum committees: Faculty insights and expectations

**DOI:** 10.12669/pjms.41.10.12430

**Published:** 2025-10

**Authors:** Afsheen Mahmood, Said Amin, Usman Mahboob, Naveed Afzal Khan

**Affiliations:** 1 Afsheen Mahmood, Department of Physiology, Khyber Girls Medical College, Peshawar, Pakistan; 2 Said Amin, Department of Medicine, Hayatabad Medical Complex, Peshawar, Pakistan; 3 Usman Mahboob, Institute of Health Professions Education, Khyber Medical University, Peshawar, Pakistan; 4 Naveed Afzal Khan, Department of Medical Education, Khyber Girls Medical College, Peshawar, Pakistan

**Keywords:** Curriculum committee, Curriculum development, Dental students, Faculty, Medical students, Perceptions

## Abstract

**Objective:**

To explore faculty insights about the undergraduate medical students’ participation in the curriculum committees.

**Methodology:**

An exploratory approach to qualitative study design was adopted. Four focus group discussions were conducted in two medical and two dental colleges of Khyber Pakhtunkhwa, based on a piloted and validated guide, over a period of six months July 2021-January 2022. The audio recordings were transcribed verbatim. Anonymity was ensured, and thematic analysis of the transcripts was undertaken using the investigator triangulation.

**Results:**

Two hundred and seven narrated codes were condensed to fifty codes after screening. Six overarching themes were derived from sixteen identified subthemes. The theme ’emotional engagement’ specifies that students feel satisfied and empowered in the curriculum development process. The theme ’cognitive engagement’ helps in gaining knowledge and critical thinking skills. The theme ’physical engagement’ indicates that the student’s physical involvement would enhance leadership skills. The theme ’Feedback and change in the educational environment’ encompasses the expected changes in the teaching environment and modes of information transfer following the incorporation of students’ suggestions. The theme Students’ stake ship in curriculum committees highlights the barriers to students’ participation in curriculum committees. The theme of ’curricular reforms’ suggests the receptive areas for the students’ participation in the curriculum and student development.

**Conclusion:**

The student’s engagement has emotional, cognitive, and physical dimensions. The reservations of teaching faculty about the students’ participation in curriculum committees have to be considered.

## INTRODUCTION

Student has been the epicenter of every educational activity.^1^ However, the voice of the students is mainly excluded from the curriculum development process.^2^ All over the world, educational advancements have considerably transformed the face of the medical curriculum and profession. Globalization has significantly impacted the process of medical and dental curriculum development, with an increasing emphasis on students’ engagement.^3^ The success of educational activities like curriculum development, teaching strategies, and program implementation depends on the collaborative approach involving all the partners to set and achieve the desired goals.^4^ The poor curriculum development process is a dilemma of developing countries, leading to program failures.^5^

In Pakistan, the process of curriculum development is expert-led. The experts are assigned the task of developing the curriculum without the active participation of all the stakeholders, such as students, faculty, and the community.^6^ After implementing new standards for the accreditation of medical colleges in Pakistan, there is growing emphasis from the accreditation authorities on the involvement of students in curriculum development activities such as curriculum planning, designing, changing, dissemination, implementation, and assessment.^7^

In Pakistan, the students have not attained the desired role in curriculum development. There are specific barriers to the engagement of students in the curriculum.^8^ The Student’s role in different domains of curriculum development and level of involvement has been the subject of debate. There are different opinions and perceptions. There is a debate about the extent to which students can be involved in educational activities.^9^ The purpose of the study was to explore the perceptions of the faculty about the students’ engagement in the curriculum committees.

## METHODOLOGY

An Exploratory Qualitative study design (Case study) was adopted for this study, which spanned over a period of six months July 2021-January 2022. Astin’s Student Involvement Theory was selected as the theoretical framework for the literature review, discussion guide development, and analysis. It postulates that the amount of student learning and personal growth associated with any educational activity, such as curriculum development, is directly proportional to the quality and quantity of student involvement in that program.^10^

A focus group discussion guide comprising 15 questions was developed, piloted, and validated to identify the different perceptions of faculty regarding students’ engagement in curriculum development, taking into account the various dimensions of Astin’s theory of engagement. Focus group discussions (FGD) were conducted in two medical and two dental colleges (one public and one private each) in Khyber Pakhtunkhwa. Purposive Sampling was used based on the experience and involvement of faculty members in curriculum development. Participants were approached after formal approval from the heads of institutes and ethical approval. Informed written consent was taken from each participant of FGD. The participants were assured of anonymity. Thematic analysis was conducted of the transcripts, incorporating investigator triangulation, and the quality of the research was ensured by adhering to the criteria outlined by Guba and Lincoln.^11^

### Ethical approval:

The Ethical Review Board of Khyber Medical University, on July 14th, 2021 (No. DIR/KMU-EB/PF/000861) issued the ethical approval.

## RESULTS

Two hundred and seven narrated codes were condensed to fifty codes after screening. Six overarching themes were derived from sixteen identified subthemes. The demographic profile and curricular experience of the participants have been mentioned in Table-II.

**Table-I:** Questions for FGD after piloting and validation.

Q1	What do you understand by the curriculum development process?
Q2	What is your concept of student participation or engagement in the curriculum?
Q3	What could be the effect of engaging the students in curriculum committees on the motivation and interest of students?
Q4	What changes do you anticipate in students’ attitudes by involving the students in the curriculum development committees?
Q5	How would involvement in the curriculum committee influence ownership of curriculum by students?
Q6	In your view, what are the barriers to the student’s participation in the curriculum committees?
Q7	How would involving students in curriculum committees influence the knowledge of students?
Q8	What could impact students’ learning by not involving the students in the curriculum development committees?
Q9	In your opinion, what are the drawbacks of involving the students in the curriculum development committees?
Q10	Would the physical presence of students in the curriculum committees improve their leadership skills?
Q11	Engaging the students in curriculum committees could impact classroom interaction and teaching methods?
Q12	Do you think the student would be able to spare dedicated time for the curricular activities?
Q13	By engaging the students in curriculum committees, what could be the impact on learning outside class
Q14	How would curriculum engagement affect the student’s role in society
Q15	Anything else you want to add on?

**Table-II:** Demographics of FGD participants.

FGD No.	College	Members (M)	Gender		Experience in curriculum (n=years)
			Male	Female	
FGD-1	MC-1	5	2	3	2-10
FGD-2	DC-1	3	2	1	2-8
FGD-3	MC-2	3	2	1	2-7
FGD-4	DC-2	3	2	1	2-5

### Theme-1, Emotional engagement:

Emotional engagement represents the energy behind the activity (negative or positive). It depends upon student empowerment and student satisfaction leading to behavioral transformation. Most of the participants of FGDs in this study were in favor of student empowerment. Student empowerment would be reflected by curriculum ownership, a sense of security, and accountability. In the eyes of most faculty members, student satisfaction was pivotal for every educational activity of the curriculum development process. Students’ interest, motivation, confidence, achievement, and attitude were the parameters identified by the faculty for student satisfaction and engagement, have been mentioned Table-III.

**Table-III:** Representative quotes for themes 1, 2 & 3.

***Faculty Quotes on Emotional engagement***
1. Member-2(DC-1): Of course, if students are involved in organizing and designing curriculum, they have higher stakes involved, and they have a sense of ownership and hence have more interest in the curriculum.
2.Member-1(DC-2): The students would feel confident because they were involved in the curriculum development. They would be responsible and accountable for the module’s content, implementation, and outcomes.
***Faculty Quotes on Cognitive engagement and change in performance***
1. Member-4(DC-1): If the students know what they are being taught, what they will be taught, how they will be taught etc., they will learn what to study, how to study it, and when to study it. They will manage their time more effectively. It will help improve student learning and knowledge.
2.Member-4(DC-1): If the students are involved in curriculum development from the start, they will naturally develop leadership skills. If the students actively give input, they would develop critical thinking and problem-solving skills beyond their age. It would help in the development of better leadership skills.
***Faculty Quotes on Physical engagement and transformation in the role***
1. Member-1(DC-1): Extracurricular activities are also a part of the curriculum. If we know that the students are interested in a particular activity, we will focus our efforts. So, I think that extracurricular activities should increase.
2.Member-1(MC-1): There are around ten members in the student committee. If you make them participate in curriculum development, teach them about it, and actively involve them in the process, you groom future leaders for society.

### Theme-2, Cognitive engagement and change in performance:

Nearly all the faculty members expressed that student engagement would improve the cognitive domain of the students. This engagement of the students would lead to enhanced learning and facilitate critical thinking have been mentioned Table-III.

### Theme-3, Physical engagement and transformation in the role:

The physical engagement in the curriculum development, along with co-curricular and extracurricular activities of students, was positively emphasized by most faculty members. This would lead to improvement of their leadership skills as well, mentioned Table-III.

### Theme-4, Feedback and change in the educational environment:

There was a consensus opinion of the faculty members that the input and feedback of the students in the curriculum development and implementation would undoubtedly impact the teaching environment and teaching methodologies. This would bring about a positive change in the classroom and institutional environment, have been mentioned Table-IV.

**Table-IV:** Representative quotes for theme 4, 5 & 6.

***Faculty Quotes on Feedback and change in the educational environment.***
1. Member-3(DC-1): Whatever we design for the student should be created from the students’ perspective. The teacher has already achieved a higher level of knowledge than the students. Therefore, to better understand the challenges faced
2.Member-1(DC-1): The classroom environment will become more interactive if the students feel heard. We would change our teaching methods based on student input, decreasing the communication gap.
***Faculty Quotes on Students’ stake ship in curricular committee***
1. Member-1(MC-3); I think the cornerstone for resistance is the faculty; we still feel that curriculum development should be faculty-centered.
2.Member-3(MC-1): If you compare our students with the students abroad, there is a clear difference in their maturity levels. Students abroad enter medical school after completing bachelor’s or master’s degrees, whereas our students enter at a younger age.
***Faculty Quotes on Curricular reforms***
1. Member-1(DC-1): The curriculum development process includes the syllabus and activities like designing, implementing, and assessing students.
2.Member-1(MC-2); I would like to endorse Member-4’s view that if you involve students in all aspects of the curriculum, then probably it’s not going to be very fruitful. Yes, if you apply them in some parts like in MITs and ask them for feedback on the difficulties they faced in the curriculum’s execution and evaluation of the curriculum.
3.Member-1(MC-2); Again, this is not fair. I feel their opinion should be taken in assessment. In some places, their views will be considered to a greater extent, and in some areas, it would be deferred altogether.
4.Member-2(DC-2): It would improve it, but everything has pros and cons. I believe that some students will always dislike the decisions. So, the final decision is always in the hands of senior faculty members on whether to listen.
5.Member-2(DC-1): If a student affiliated with a political party is involved in this process, the process would be compromised. So, students interested in these committees must not be active workers of any political party.

### Theme-5, Students’ stake ship in curriculum committee:

There was some resistance among the stakeholders about the share and autonomy of students in curriculum development. The teaching faculty conveyed their reservation about the student’s participation and independence. Concerns regarding students’ maturity and developmental stage were identified as barriers to their meaningful engagement, have been mentioned Table-IV.

### Theme-6, Curricular reforms:

The participants had mixed opinions about the involvement of the students in the curricular reform process, especially those students with active affiliations with political parties and their student associations. The majority were in favor of including them in decisions regarding teaching strategies and incorporating their feedback during the curriculum evaluation process, have been mentioned Table-IV.

## DISCUSSION

The student engagement in curriculum committees in the curriculum development process has been multi-dimensional. Student engagement has been linked with favorable academic achievements, improved attendance, and better mental health. For the best outcomes of curricular activities, the Student’s emotional, cognitive, and emotional engagement is paramount.^12^ Emotional engagement in curriculum refers to the negative and positive reactions to the educational activity. It is measured by Student empowerment (ownership, sense of security, accountability) and student satisfaction (interest, motivation, confidence) in curricular activity.^13^

In the current study, the teaching faculty believed that student empowerment in curricular committees would undoubtedly improve their sense of ownership and security, best reflected by the quote, ’When the student is actively involved in the construction of something, they develop an attachment with that thing. The same observation has been reported by Christenson et al. and Patrick et al.^14^,^15^ Student satisfaction with curricular activities, reflected through interest and motivation, has been reported in the literature by Linnenbrink-Garcia et al. from Texas, United States.^16^

Cognitive engagement in the classroom can be characterized as a psychological state in which students put in a lot of effort to truly understand a topic and in which students persist in studying over a long period.^17^ Student participation in curricular activities positively impacts the students’ learning and fosters critical thinking. These findings have also been observed by Arnold and Ritchie from the USA.^17^,^18^ The current study emphasized the physical engagement of the students and the development of better community leaders. Blomfield and Barber also observed that participating in extracurricular activities adds to leadership talents and character growth.^19^

The participants reported that student feedback would transform the teaching environment and significantly impact the modes of information transfer. They also expressed concerns about the faculty preparedness and development for such modifications. This phenomenon has also been witnessed by Davies et al.^20^ The current study findings revealed diverse perceptions among teaching faculty regarding student participation in curriculum development. The traditionally trained faculty members expressed many reservations about student engagement at every step of curriculum development. These findings were not different from the reports of Sturtevant and Wheeler, where the faculty showed an unwillingness to change the traditional approach.^21^ The faculty expressed their reservations about the students’ immaturity and questioned their knowledge about the curriculum development process. This notion has been noted by Sanchez et al.^22^

Some of the faculty members were also concerned about the political influences and intrusion of student unions and organizations, especially in the student selection process for student representation in curriculum development. Goodell and Yusko have also highlighted similar issues.^23^

The current study reports the numerous benefits and positive influences that student engagement in the curriculum development will bring about. Despite some reservations, the faculty strongly advocated for the inclusion of students in the curriculum development process.

### Strengths:

This study’s strength was the use of Astin’s Student Involvement Theory as the theoretical framework for the literature review, discussion guide development, and analysis. This approach to qualitative study facilitated a rigorous exploration of complex issues and key emerging themes.

### Limitations:

This study represented only the perspective of the teaching faculty about student participation in the curriculum development and the curriculum committee.

## CONCLUSIONS

The emotional engagement, empowerment, and satisfaction of students will be enhanced by their involvement in curriculum committees, according to the teaching faculty. The student’s physical involvement will contribute to an increase in the development of leadership skills, enhance critical thinking, and increase knowledge. The faculty believed that the teaching environment, particularly the teaching methodologies, would be transformed by the students’ involvement. The reservations of teaching faculty about the students’ participation in curriculum committees also have to be considered.

### Future direction:

Future research should explore the processes of student selection, the extent of student autonomy in curriculum development, and administrative perspectives on facilitating student participation.

### Author’s Contribution:

**AM & SA:** Conceived, designed, did data collection, data analysis, and manuscript writing, and are responsible for the integrity of the research.

**UM:** Designed, analyzed the data, reviewed, and approved the manuscript.

**NAK:** Was involved in design, data analysis, editing, and manuscript writing.
